# Can an Emoji a Day Keep the Doctor Away? An Explorative Mixed-Methods Feasibility Study to Develop a Self-Help App for Youth With Mental Health Problems

**DOI:** 10.3389/fpsyt.2019.00593

**Published:** 2019-08-23

**Authors:** Levi Van Dam, Sianne Rietstra, Eva Van der Drift, Geert Jan J. M. Stams, Rob Van der Mei, Maria Mahfoud, Arne Popma, Eric Schlossberg, Alex Pentland, Todd G. Reid

**Affiliations:** ^1^Spirit Youth Care Amsterdam, Amsterdam, Netherlands; ^2^Department of Child Development and Education, University of Amsterdam, Amsterdam, Netherlands; ^3^Department of Antropolgy, Utrecht University, Utrecht, Netherlands; ^4^Centrum Wiskunde and Informatica, Amsterdam, Netherlands; ^5^Department of Child and Adolescent Psychiatry, Amsterdam UMC/De Bascule, Amsterdam, Netherlands; ^6^Institute for Criminal Law and Criminology, Leiden University, Leiden, Netherlands; ^7^Connection Science, Massachusetts Institute of Technology, Cambridge, MA, United States; ^8^Media Lab, Massachusetts Institute of Technology, Cambridge, MA, United States; ^9^Harvard T. H. Chan School of Public Health, Boston, MA, United States

**Keywords:** ecological momentary assessment, youth at risk, emojis, mobile health interventions, adolescence

## Abstract

Today’s smartphones allow for a wide range of “big data” measurement, for example, ecological momentary assessment (EMA), whereby behaviours are repeatedly assessed within a person’s natural environment. With this type of data, we can better understand – and predict – risk for behavioral and health issues and opportunities for (self-monitoring) interventions. In this mixed-methods feasibility study, through convenience sampling we collected data from 32 participants (aged 16–24) over a period of three months. To gain more insight into the app experiences of youth with mental health problems, we interviewed a subsample of 10 adolescents who received psycthological treatment. The results from this feasibility study indicate that emojis) can be used to identify positive and negative feelings, and individual pattern analyses of emojis may be useful for clinical purposes. While adolescents receiving mental health care are positive about future applications, these findings also highlight some caveats, such as possible drawback of inaccurate representation and incorrect predictions of emotional states. Therefore, at this stage, the app should always be combined with professional counseling. Results from this small pilot study warrant replication with studies of substantially larger sample size.

## Introduction

In today’s society, mobile technology allows people to be in and out of contact with each other seamlessly and continuously. Currently, in the Netherlands, 98.2% of the young people between 12 and 24 years of age have a mobile phone to access the Internet (1). With this being such an important medium for young people, even partially substituting in-person contact with the technology (2) in youth mental health care might be an effective intervention or contribute to the effectiveness of youth psychological treatment (2–5). A recent meta-analysis on the effectiveness of mobile health as a supplement to mental health interventions for youth suggests that the mobile phone may enrich youth therapy (6). Mobile supported therapy of shorter lengths yielded larger effects for treatment adherence and weight-management.

Weisz et al. (7) conducted a multi-level meta-analysis on youth psychological treatment outcomes over the past five decades. Significant positive treatment effects were found for anxiety (medium effect) and depression (small effect) but not for youth with multiple problems (7). To enhance therapeutic effects for those with complex needs, the authors propose extending treatment to youth’s everyday lives and personalize treatment through the implementation of add-ons, such as an additional drug therapy, wireless devices, and/or more traditional supplemental interventions (7, 8).

In this explorative mixed-methods feasibility study, we describe the development of G-Moji, an mHealth intervention in which “the technology aims to enhance treatment or assessment, increase dissemination of interventions, or provide clinicians and clients with greater choice for accessing treatment materials or activities” (9, p. 1). Advantages of technologically enriched treatments are the possibility of reducing costs, giving the clients an active role in their treatment process, and making greater impact.

A new way to assess mental health problems is by using a technological form of measurement, designated as ecological momentary assessment (EMA), whereby behaviours are repeatedly assessed within a person’s natural environment (10). This form of measurement is promising because it enables more accurate daily measurements compared to questionnaires administered intermittently, it makes it feasible to provide personal advice, and it may detect mental health problems at an early stage. The latter makes it possible to shift the focus more from treatment to prevention and aims to help empower youth through self-monitoring.

Communicating mental health issues can be very challenging, especially for teenagers and early adolescents (11). Emojis, from the Japanese *e* [picture] + *moji* [character] are graphic symbols, such as 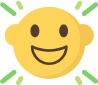. They offer a new way of communication about emotions, mood, and physical state, with the benefit that these emojis are already well integrated into the daily lives of individuals through the ubiquitous use of digital devices and social media. Emojis are the innovative form of emoticons (a portmanteau of “emotion” and “icon”, that use punctuation to depict emotions, i.e., :-)), and they use vivid pictographs of faces, objects, and symbols. However, studies warrant caution interpreting emoticons and emojis, especially given that cultural differences might lead to different interpretations of similar emojis (12). There also exist gender differences. Girls prefer using emojis more than boys (13). Despite these differences, however, emojis could prove to be helpful with youth by allowing them to communicate their mental health state and better understand the challenges with managing their health (14). To our knowledge, however, differences between youth with and without mental health problems and their interpretation or use of emojis have not yet been explored.

Our pilot study combines questionnaires, ecological momentary assessment, and interviews to explore the feasibility of a new mHealth self-monitoring tool as an intervention to empower youth with mental health problems. To conduct this study, a new app called “G-Moji” was developed.

The present study first explored the frequency of emoji use and whether these emojis were perceived as negative and positive emotions by the participants. Second, we aimed to identify differences in self-report of negative and positive emojis between a group of adolescents receiving youth care and a non-clinical comparison group of adolescents from the general population. Third, we examined whether report of negative and positive emojis were associated with mental health problems (i.e., psycho-neuroticism) and resilience. After these group level analyses, we conducted individual pattern analysis in order to examine if patterns of emoji use over a three-month period were different in two participants from the “clinical” youth care group and the comparison group. Different patterns would support the use of emojis for clinical purposes in order to be able to fine-tune interventions from the perspective of personalized treatment. We also interviewed a subsample of the participants receiving treatment to explore their experiences and perspectives on the potential advantages and disadvantages of the emoji-driven app. Results of this study will contribute to the current knowledge of mHealth interventions, since this is the first study that examines this type of intervention for youth with complex needs.

## Method

### Participants

The study included 32 participants between 16 and 24 years of age (*M* = 20.06, *SD* = 2.54), 78% were female and 84.4% of Dutch ethnicity. Of the participants, 41% (*n* = 13) received mental health care from a municipality service, mental health care ranging from mild (e.g. psychological counselling) to severe (e.g. residential treatment). Within this specific group, the average age was 19 (*M* = 18.85, *SD* = 2.51), 78% were female, 77% of Dutch ethnicity, and 55% had education beyond high school. The average age within the group not receiving youth care (*n* = 19) was 21 (*M* = 20.89, *SD* = 2.26), 79% female, 90% of Dutch ethnicity, and 71% with education beyond high school.

A subsample of *n* = 10 participated in the qualitative study, aged between 16 and 22 (*M* = 18.5, *SD* = 1.86). Of this subsample, 70% were female 76.9% of Dutch ethnicity, and they all received some type of psychological support ranging from mild (psychological counselling) to severe (residential treatment).

### Procedure and Exclusion Criteria

Convenience sampling was used to recruit participants: healthy participants were recruited through snowball sampling, and youth receiving mental health care were recruited from de Bascule, a child and adolescent psychiatric facility. Participants were met in person at a location of their choice. The goal of the study was explained and questions answered. To make certain that every participant was aware of their rights and our privacy statement, they all signed an informed consent. Participation was voluntary and termination was possible at all times. As a reward, the participants received a power bank for their wireless devices along with € 5 for each month of participation. Inclusion and exclusion criteria were based on the type of smartphone operating system, residence of the participant, and whether their smartphone use was work-related or for personal use. The G-Moji app is only available for Android, so participants with other operating systems (e.g., iOS) were excluded. For practical reasons, it was decided to exclude the participants with a work phone, because they would not be able to answer the questions daily, and data could only be collected five days a week during day-time hours. The data collection lasted three months. Since the app is developed for youth with mental health problems, we randomly selected a subsample of the clinical population to gain more insight in their experiences through in-depth interviews. After the completion of the three months, participants decided if they wanted to keep using the app or uninstall it from their phones.

## Measures

### Data Collection Through Smartphone — Continuously Throughout the Three-Month Period

**Figure 1:**
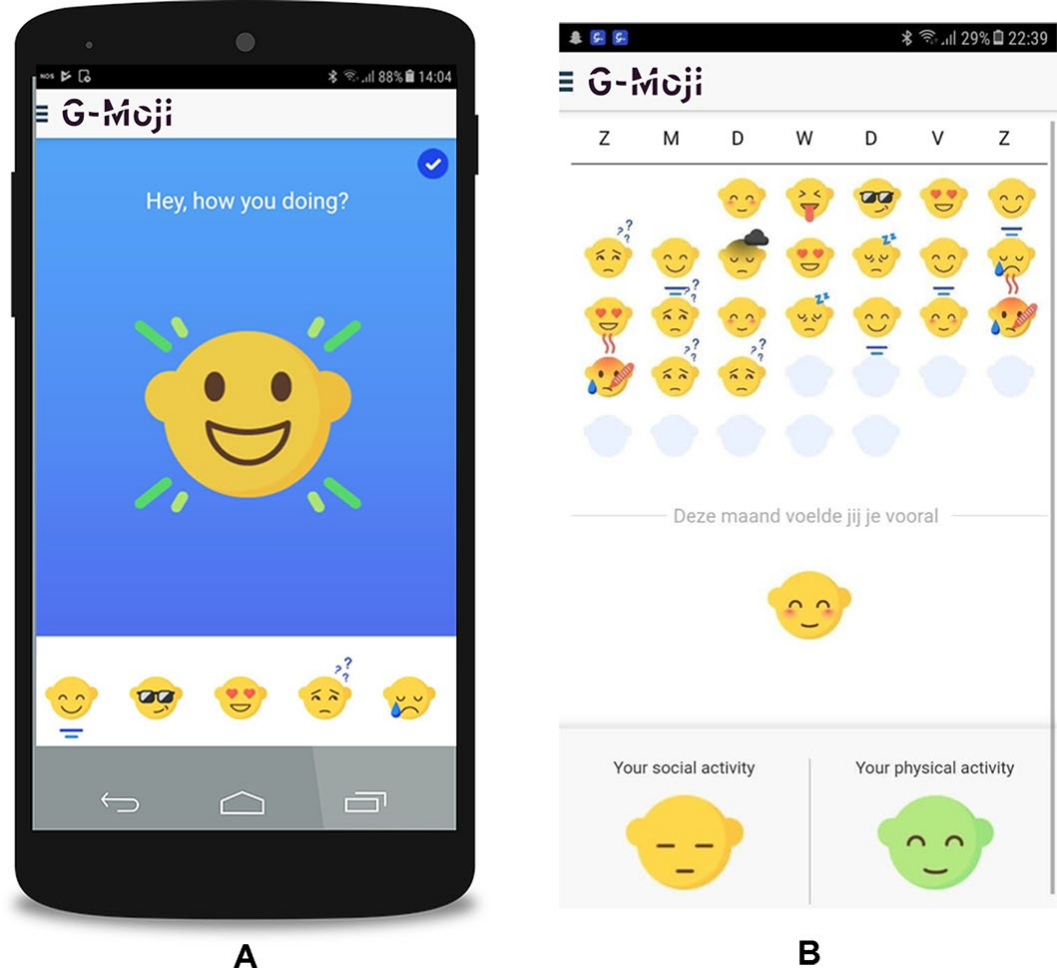
Screenshots of G-Moji app used for collecting self-reported feelings. **(A)** Daily question to answer with a emoji (ecstatic). **(B)** Monthly overview of selected emojis which also gives an overall feeling of the month and shows the social and physical activity level of the participant.

Participants used the G-Moji app (**Figure 1**), which is currently in its developmental stage. Feedback from the participants will be used to further develop the app. At the beginning of the evening, the “G-Moji” app asked one daily short survey question: “How are you feeling today?”. Participants responded by selecting one out of fourteen emoji icons to describe the following feelings: anxious, confident, confused, down, ecstatic, funny, happy, hopeless, love, mad, peaceful, sad, sick, or tired. Emoji icons were used, because this is a natural, attractive and easy way for adolescents to respond. Moreover, the G-Moji app also collects socio-behavioral passive data (e.g., call logs, Bluetooth devices in proximity, cell tower IDs, application usage, and phone status, such as charging and idle) to infer a) activity levels, b) social interactions (how frequently they interact with whom in their network), c) sleep and d) general routineness. Because of the scope of this study, these data are not taken into account yet.

### Questionnaires — Pre- and Post-Measurement

Physical and psychological symptoms. The Symptom Checklist (SCL-90, Dutch version) was used to assess if the participants had any physical or mental health issues. The self-report checklist contains 90 statements based on a five-point Likert-scale of distress, ranging from “not at all” (1) to “extremely” (5). The checklist contains eight subscales; agoraphobia (AGO), anxiety (ANX), depression (DEP), somatization (SOM), inadequacy of thinking and acting (IN), interpersonal sensitivity (SEN), hostility (HOS) and sleep issues (SLE) (15). Next to these scales are nine non-scaled items with questions about eating disorders and psychoticism, which contribute to the total score of psycho-neuroticism (PSNEUR). The General Severity Index (GSI) displays the average score and provides an overall measure of psychiatric distress (15). The total score of psycho-neuroticism ranges from 90 to 450; individuals with a score equal to or higher than 224 are highly likely to experience some kind of psychopathology.

The SCL-90 is widely used as an assessment instrument for the screening of mental health problems and evaluation of treatment results. The psychometric properties have been widely investigated and were found to be satisfactory. The internal consistency of the scales range from .77 to .90 (16), which means that all scales can be qualified as excellent according to the margins of Cicchetti (17). The test–retest reliability ranges from .68 to .90 (18, 19).

In this study, the Cronbach’s alpha reliabilities were .97 at pre-test and .99 at post-test. The pre- and post-test scores were significantly correlated (*r* = .83, *p* < .001). Based on this correlation, and because participants reported their emotional states by means of emojis between pre- and post-test, we decided to compute an average psycho-neuroticism score. This was not normally distributed and it showed a substantial positive skewness. As a result, we log-transformed the overall score to obtain normality. We did not find outliers, based on criteria formulated by Tabachnick and Fidell: –3.29 < *z* < 3.29 (2013).

*Resilience.* The Child and Youth Resilience Measure (CYRM-12) was used to assess the resilience of the participants. The questionnaire consists of 13 basic questions about education and residency, and contains 12 items based on a five point Likert scale, ranging from “not at all” (1) to “extremely” (5). These items measure individual capacities, relationships with primary caregivers, a sense of social support, and account for diverse social contexts across cultures.

The validation of the CYRM has been investigated in different countries for both English and translated versions. The reliability of this questionnaire is sufficient (α = .84) (20). The Dutch version has not been extensively validated, but the questionnaire has been designed to be culturally sensitive. It showed positive psychometric properties in a recent general population study among youth from Curaçao in that the original factor structure was replicated and proved to be measurement invariant across Dutch and Papiamento speaking youth, age, and gender, while reliability proved to be satisfactory (21).

In the present study, Cronbach’s alpha reliabilities were .76 at pre-test and .74 at post-test. The pre- and post-test scores were significantly correlated (*r* = .65, *p* < .001). Based on this correlation, and because participants reported their emotional states by means of emojis between pre- and post-test, we decided to compute an average resilience score, which proved to be normally distributed. We did not find outliers, based on criteria formulated by Tabachnick and Fidell (22). Because no valid cut-off scores are available in order to establish which score represents the boundary between the “normal” and “clinical” range, we created (pre-test and post-test) percentile scores for the present sample in order to facilitate comparisons at the individual level.

### Interviews — During Participation

In addition to questions about their experiences with the app, participants were also asked to reflect on the growing trend of “datafication of health”: the representation of many aspects of life as quantified data (23). They were asked about the potential risks of this development (e.g., a situation involving elevated odds of undesirable outcomes), and resilient factors (e.g., the process of harnessing key resources to sustain well-being) (2015). Interviews were conducted using an semi-structured interview approach based on a pre-formulated topic list.

### Quantitative Analysis

Analyses were conducted without data imputation to compensate for missing values. First, a descriptive analysis to examine the frequencies of the 14 emojis was conducted. This led to the exclusion of the emoji “hopeless”, since it was never chosen. Subsequently, we conducted a principal component analysis with oblimin rotation for correlated factors, with a forced two-dimensional solution, in order to establish whether a distinction could be made between a negative and positive dimension in experiencing emotional states by means of self-report through emojis, using absolute instead of relative frequencies. Next, we examined whether youth receiving care experience more negative emotions and less positive emotions, less resilience and more psycho-neuroticism than youth from the comparison group by means of a series of t-tests. Finally, we examined correlations between the negative and positive emojis and also psycho-neuroticism and resilience by computing simple Pearson’s correlation coefficients.

### Qualitative Analysis

In-depth readings of the complete interview transcripts were conducted. The qualitative data analysis software program NVivo was used to develop a codebook, based on the two thematic areas of the topic list: (a) risks and (b) resilience. Initial themes were identified by the third author and verified by the first author, using the iterative thematic approach from Boeije (2005), following guidelines as formulated by Tong et al. (24) to secure the validity and reliability of qualitative study findings. During the initial coding phase (Step 1), we reviewed the transcripts to identify emerging themes, based on the initial codebook. Next, we noted possible relations between codes and groups and developed descriptive codes and categories (Step 2). We then conducted our final analyses by reviewing the code clustering (Step 3). The first author served as master coder, reviewing the work of and providing feedback to the coder to ensure consistency in coding across cases. Transcription and data analysis were in Dutch, with key quotes translated into English. Further details about the design and method of the study can be obtained with the first author.

## Results

### Quantitative Study

In total, the participants reported 2,217 emojis during the 3 months (90 days) of data collection. The number of times a given participant selected an emoji varied from 1 to 146, the average response rate was 67%, while the median was 77%. The app was most intensively used during the first 30 days (42% of all responses), with a gradual decline in the second 30 days (33% of all responses), and the lowest response rate in the final 30 days (25% of all responses). A total of 63% of the participants did use the app during the whole 3-month period, with short time lapses of 1 or 2 days. Two participants stopped using the app after the first day. Survival analysis showed that the average time until premature termination of the emoji application was 72 days, with only marginal differences for age, gender and clinical status, which did not reach significance. **Table 1** describes the variation in the frequency of the different emojis. Upon inspection, **Table 1** shows that happy, peaceful, and tired had the highest frequencies, whereas funny, love and mad had the lowest. In addition, the participants reported more positive (60%) than negative (40%) emotions.

**Table 1:** Frequencies of emojis (*N* = 32).

	Maximum	M	SD
Happy	38	13.62	12.52
Peaceful	36	12.31	8.18
Tired	33	11.09	9.10
Ecstatic	47	6.31	9.22
Confused	19	5.44	5.32
Down	22	4.60	6.42
Confident	26	4.13	6.19
Sad	11	3.03	3.34
Anxious	17	2.56	3.77
Sick	10	2.22	2.99
Love	10	1.72	2,16
Funny	19	1.16	3.42
Mad	10	1.09	1.94

A principal component analyses (PCA) within-subject selected emojis over time, with oblimin rotation and factor loadings of .40 as a cut-off criterion, yielded a positive and negative dimension, which was consistent with our expectations. The emoji “sick” did not meet the .40 cut-off criterion and was therefore removed from the PC-analysis, loading .20 on both dimensions. Notably, “sick” might not be perceived subjectively as a negative psychological state (i.e., negative emotion) given the presence of a thermometer in the emoji but as an objective negative physical state instead. The two dimensions, which consisted of six items each, accounted for 50% of the total variance (**Table 2**). Internal consistency analyses revealed that the scale for positive emotions was only marginally reliable, showing a low standardized Cronbach’s alpha of .53 (Guttman’s Lambda 2 was .55), whereas the scale for positive emojis proved to be reliable, with a standardized Cronbach’s alpha of .87 (Guttman’s Lambda 2 was .88). The scale for positive emojis showed a normal distribution, without outliers. Also, the scale for negative emojis did not have outliers, but it showed a moderate positive skewness and was therefore changed to normal by means of a quadratic transformation.

**Table 2:** Principal component analysis of emojis.

	Component	
	1	2
Down	.844	
Confused	.822	
Mad	.792	
Anxious	.791	
Sad	.742	
Tired	.602	
Ecstatic		.605
Confident		.590
Happy		.559
Peaceful		.491
Love		.490
Funny		.479

Extraction method; principal component analysis; Rotation method; Oblimin with Kaiser normalization.

Unexpectedly, the two dimensions were positively correlated (*r* = .35, *p* = .05), showing a trend to indicate that participants who select more negative emojis also select more positive emojis and vice versa. However, if corrected for the frequency of selecting emojis, the dimensions showed a negative and significant correlation (*r* = –0.66, *p* = < .001), indicating that participants who select more negative emoji’s also select less positive emojis.

Participants receiving youth care had significantly higher scores on psycho-neuroticism (*t* = –4.494, *df* = 30, *p* < .001 and Cohen’s *d* = 1.70) and lower scores on resilience (*t* = 1.762, *df* = 30, *p* = .044 and Cohen’s *d* = 0.63) than participants from the comparison group, indicating that participants with youth care reported substantially more psychological dysfunction and less resilience. No differences were found with positive or negative emotions, although the results for the positive emojis were in the expected direction (Cohen’s *d* = 0.24), which was not true for the negative emojis, but again the difference proved to be small (Cohen’s *d* = 0.24), indicating participants with youth care reported less positive emotions (expected direction), but also less negative emotions (not in the expected direction). No different results were obtained when the analyses were repeated with the 13 separate emojis, even without correction for multiple testing.

Finally, the correlations between negative and positive emotions and also between psycho-neuroticism and resilience on the other hand ranged between *r* = –0.003 (*p* = .985) and r = –0.076 (*p* = .678), respectively. Repeating the analyses with the 13 separate emoji’s, with and without chance correction, did not yield significant results either (*p* > .10, without chance correction). However, correlations were higher now (–0.012 < r < –0.295), but still small or even very small, and not always in the expected direction.

Notably, all analyses were conducted on the frequencies of emoji use. We repeated all analyses by using the proportions of emoji of each participant (i.e., the number of times an emoji is selected as a proportion of the total frequency of emoji selection), which did not yield an interpretable factor solution in the PC-analyses. In addition, analyses based on proportions showed similar (non-significant) results when comparing youth with and without youth care and in the correlational analyses on single emoji use if compared with results from the analyses that were based on frequencies of emojis.

**Figure 2:**
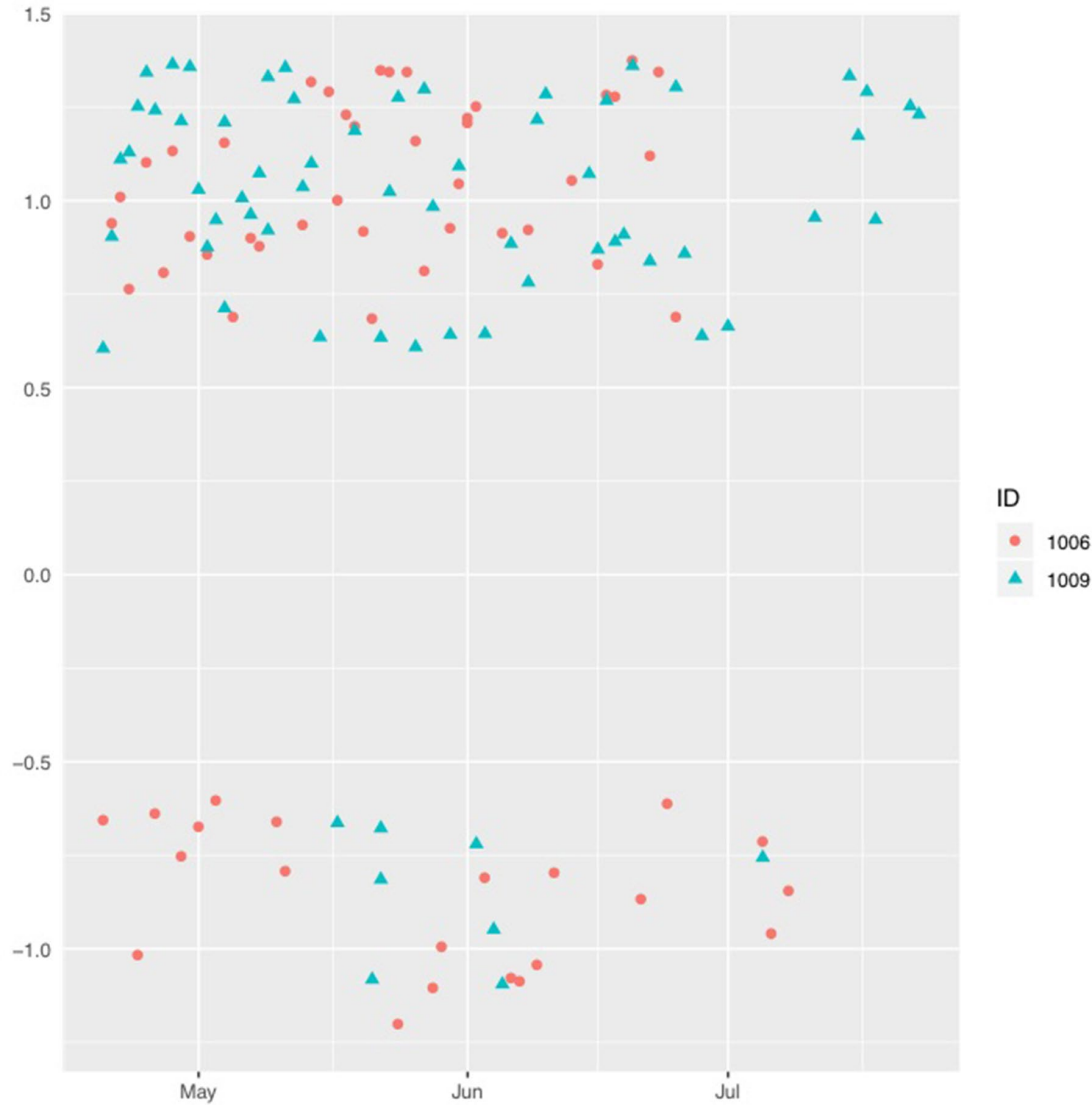
Reported positive (0.5–1.5) and negative (–0.5– –1.0) emojis during the study.

An individual case comparison was made to identify possible different patterns between two participants with an almost similar frequency of emojis, one from the youth care group (participant 1006; 61 emoticons) who attempted a suicide at the beginning of June and one from the “healthy” comparison group (participant 1009; 65 emoticons). **Figure 2** shows the reported positive and negative emojis over the three month period (May until July) for the participant from the youth care group (SCL total scores of 369 at pre-test and 395 at post-test, representing the clinical range, and a CYRM total score of 51 at pre-test and 43 at post-test, which is at the 72^nd^ and 38^th^ percentile, respectively) and for the participant from the comparison group (SCL total score of 107 at pre-test and 95 at post-test, representing the normal range, and a CYRM total score of 48 at pre-test and 46 at post-test, which scores are both at the 50^th^ percentile). The data was not transformed, but visualization was improved by adding some minor random error through jittering.

During May and June, the participant receiving youth care (1,006) consistently reported positive and negative emotions, whereas in July only negative emotions were reported. The participant without youth care (1,009) reported several negative emotions at the end of May and the beginning of July, whereas positive emojis were reported during the whole study period. Both participants reported more positive emotions than negative emotions.

### Qualitative Study

In this part, we describe the findings in detail regarding the two thematic topics: a) resilience and b) risks. The results are organized in **Table 3** to give an integrative image, after which they are described in more detail.

**Table 3:** Key themes of the qualitative study.

**Resilience**	*Increase of self-awareness:* increased awareness about behavioral patterns that motivate to change lifestyle positively, giving a sense of control.	*Personalized care:* receive tailored information from the app, based on the predictive function.	*Autonomy:* flexibility in blending face-to-face meetings with online support.
**Risk**	*Inaccuracy in prediction:* concerns about the accuracy and reliability of the prediction of mood.	*Privacy:* data is unsafe on the Internet, this app would not be less safe than other apps.	*Being controlled by an app:* these mobile health technologies might result in youth relying on apps instead of their own feelings.

The results should be interpreted with caution, because it is based on a small number of participants who may not be representative for the population of youth using similar apps with the same purpose.

Three resilient factors were identified: 1) increase of self-awareness, 2) personalized care, and 3) autonomy. For self-awareness, all participants argued that mobile health technologies have the potential to increase awareness about their behavioral patterns and motivate them to change their lifestyle in favor of their wellbeing. They stress that this type of app could give them a sense of control, and has the potential to confront them with how they are really feeling. *“Most of the times I do not pay attention to how I was feeling over the month, but now you can do something about it, because the app shows you the overview”*(James, #16) However, for youth with severe mental health problems, for example struggling with self-harm, this is difficult. Kim (20) explains that she feels empty if she has a hard time identifying her current emotion, at such moments* “it might be helpful if the app could give me a suggestion with how I’m feeling, such as you could be sad or angry”*. During the pilot, Kim’s self-harm problems became so intense, that she was referred to a residential crisis facility. In her crisis, she stated: *“I quit choosing emojis, because my head is too full with different emotions”*. Most youngsters replied that they did not consider their feelings more than usual by tracking their emotions in the research.

Regarding personalized care, all youngsters prefer to receive tailored information from the future version of the app, and most stress that besides the predictive function, the app should also be able to provide personalized advice. Julia (19), for example, illustrates that the app might help her with putting her fears into perspective, because she finds this difficult to do on her own. *“Normally I ask my friends if my fear is qualified in a certain situation. However, they are not always available, and I feel like a burden if I’m always talking about my problems. Asking an app for advice would be great”*. Many youngsters stress that the next version of the app could help them reach their goals. All youngsters considered it important to customize the evolved app according to their wishes.

Concerning autonomy, youngsters mostly mentioned flexibility in blending face-to-face meetings with online support. They did not think mobile health applications should substitute social workers completely; they prefer blended therapy. Jade (20) stated: *“You can ask SIRI, but then you get weird answers, not a real conversation. Furthermore, a social worker can help you with self-reflection, an app can’t do that of course. A social worker can meet your needs, an app can’t. Or it becomes really scary. No, let’s not do that”*. However, they are convinced that an app could offer support, especially during the waiting list period. Fleur (22), for example, was put on a waiting list for intensive trauma therapy and needed to wait another ten weeks. *“I need a crisis time out, but now I need to wait for another two months. You wait and survive. An app is at least something if you don’t have any support at all. It is not much, definitely not a human, but it might help.”*

Additionally, three risk factors were identified: 1) inaccuracy in prediction, 2) privacy and 3) being controlled by an app. Regarding prediction inaccuracy, some participants expressed concern about the reliability of a future version of the app by speaking about the inaccuracy of other devices and applications they had used. For example, Fleur (22) used two apps simultaneously to track her steps and discovered a big difference in the results of both apps. The perceived unreliability of apps raises questions about accuracy of the prediction of feelings. Therefore, the future app must be scientifically validated in order to be sure that the prediction of mood is correct, because an inaccurate prediction could result in bad feelings. As Jade (20) explains: *“if an app says you are sad or you are going to be sad, you might interpret this feedback as the feeling that you should have and as a result you will feel sad, even though the prediction might be wrong”*. However, it also depends on your current mood for how negatively a wrong prediction is perceived, as Julia (19) illustrates: *“I wouldn’t mind a wrong prediction much if I’m feeling really happy, but if I’m on the edge it might make me feel a bit sadder because it makes me doubt my happy mood”*. David (18) thinks this could also work in the other direction: *“if you are depressed and your phone says that you are super happy, then it will actually go worse”*. Therefore, youngsters stress that in some cases an inaccurate prediction could become very risky, and they worry that it might even become fatal for youngsters with suicidal thoughts. Consequently, some participants stressed that the future version of the app should not become completely predictive. Instead, the user should be given the possibility to fill in the right emoji themselves if the application predicts their mood wrongly. Two participants, Carmen (23) and Fleur (22), had neurotic symptoms (assessed with the SCL-90) and suggested that giving user input might become another compulsion for youngsters with neurotic tendencies.

Considering privacy, these youngsters believe that their data is unsafe anyway on the Internet, and that the research and future app would not be less safe than other apps. Therefore, these youngsters did not care much whether their data was being sold to third parties. David (18) for example, was not worried about his privacy on a self-tracking app, *“since the app only has unimportant information like my profile picture, weight, length and heartbeat”*. Julia (19) shares art on Instagram and follows tattoo artists.* “I think it is really innocent, so I wouldn’t be scared if my information would be shared [with third parties] or something like that, because there isn’t something interesting anyways”*. Most participants thought their collected data would not be important enough or that could be used in a harmful way by thirds parties.

As for being controlled by an app, youngsters stressed the controlling effects of mobile health technologies. Fleur (22), for example, was concerned that users of mobile health applications might only listen to their app instead of their own feelings: *“it is certainly a danger that emojis generated by the computer might determine our real-life emotions”*. David (18) was firmly opposed to the a future version of the app: *“it annoys me that a computer would tell me how I’m feeling, of course I know this better than an app. Emotions are what distinguishes a human from a robot and if an app is acting like he is the boss about your emotions by predicting your mood, you are no more than a robot”*.

Apart from these possible advantages and disadvantages, the desirability of interaction through a chat function in the app with other app-users was investigated. All participants, apart from James (17), would not use this chat function that would connect them with other (at-risk) youth, because they were not interested in meeting new people. However, they thought that other youth would like to have the ability to share their story with other users. Therefore, this chat function should be optional, so that youth experiencing similar issues might support each other. However, they indicated that this could also go wrong, since adolescents might assist each other in planning dangerous activities, such as suicide attempts.

## Discussion

The aim of our mixed-method study was to investigate whether the use of emojis is feasible for research purposes, providing a new assessment method for acquiring knowledge on the aetiology of mental health problems of adolescents with complex needs receiving youth care, and as a clinical tool that can be used for self-monitoring, in particular as an add-on to regular treatment.

Although two emojis were excluded (hopeless and sick), the other 12 emojis represented negative and positive emotional states, with overall more positive (60%) than negative (40%) feelings. No differences were found in self-report of negative and positive emojis between youth from the “clinical” group and comparison group, while negative and positive emojis were not associated with mental problems and resilience. However, individual case analyses did reveal (clinically meaningful) different patterns of emoji use over a three-month period between a participant from the youth care group, scoring in the clinical range on psycho-neuroticism and showing a sharp decrease in resilience from pre-test to post-test, and a participant from the comparison group scoring in the normal range on psycho-neuroticism and average resilience. Given that principal component analyses of the 12 emojis yielded two well-interpretable dimensions of negative and positive emotions and the clinically meaningful individual differences in patterns of emoji use, further research on the emoji app in clinical practice seems warranted.

The qualitative part of our study revealed that through this type of mHealth intervention, youth experienced an increase of self-awareness and autonomy and see opportunities for personalized care. Nevertheless, they are concerned about inaccurate representation and prediction of emotional states, privacy, and the idea of being controlled by an app. Connecting youth with mental health problems with each other through a chat function on the app may facilitate mutual support, but was also evaluated as risky by the participants, since this could lead to planning harmful activities together, such as suicide attempts.

The fact that the principal component analysis of the emojis yielded two well-interpretable dimensions seems important, especially because emojis are relatively independent from technology developments. Current touch screens, for example, might soon be replaced by eye-tracking or gesture based interfaces, each technology development requiring new studies to interpret this new type of data (25). Emojis, on the other hand, might offer a relatively stable part of smartphone usage. Although studies warrant caution interpreting emojis, especially since cultural differences might lead to different interpretations of similar ones (12), none to date have investigated differences in interpretation of emojis between youth with and without mental health problems. Therefore, our results from the principal component analysis warrant replication with a substantially larger sample in order to be able to conduct multi-group confirmatory factor analysis, examining measurement invariance between the clinical and non-clinical group, different ethnic groups, sex and age.

The emojis “sick” and “hopeless” should be excluded in future studies. The emoji “sick” might not be perceived as a subjective negative psychological state (i.e., negative emotion) given the presence of a thermometer in the emojis, but as an objective negative physical state. The emoji “hopeless” was not reported. Future studies could enrich their emojis with the Lisbon Emoji and Emoticon Database, which divided 153 emoji in seven dimensions for emojis from iOS, Android, Facebook, and Emojipedia (26).

The SCL-90 has been developed for valid and reliable assessment of psycho-neuroticism at both the individual and group level, with high levels of specificity and sensitivity; and, thus, low chance of false positives and false negatives. Notably, there is an ongoing discussion about the validity, reliability, and usefulness of group level research, because of large individual differences among youth receiving treatment for complex needs. Nevertheless, our data show that the SCL-90 has great predictive power with regard to the discrimination between the clinical and non-clinical comparison group, both at the group and individual level. As we are conducting a feasibility study, it seems important to use the SCL-90 in subsequent research on the G-Moji. The combination of different assessment methods, such as retrospective evaluations by means of questionnaire self-report (SCL-90) and daily (momentary) self-perception of emotional states through a mobile device (G-Moji), lead to a more elaborate and integrated assessment of adolescents’ mental health (27).

Our study has several limitations, which are primarily associated with the explorative character of our feasibility study, such as convenience sampling and a small sample size, resulting in little statistical power and limited external validity. Most participants did not use the app every day, which made it difficult to compare patterns of emoji use at the group level. Our individual comparison is for illustrative purposes and needs further statistical elaboration in future research, statistically testing profiles after cluster analyses. In doing so, future research may reveal clinically meaningful differences in patterns of emoji use between groups of adolescents with and without mental problems over longer periods of time by using Generalized Linear Mixed Models and Cluster analysis. We could not reliably distinguish between youth receiving psychological treatment and youth from the normal comparison group on the basis of frequencies of emoji use at the group level. Notably, the emoji app has been designed to assess the dynamics of daily changes in emotional states over a longer period of time, and it is therefore plausible to suggest that future group level analyses of such individual differences might reveal that different patterns of emotional states shed more light on the aetiology of mental problems in youth with special needs, providing new tools for effective personalized treatment. Time-of-day effects should be taken into account in future studies, since previous studies indicate differences in responses result from the moment during the day in which a question is asked (28). Future studies should compare the use of emojis on communication platforms youth already use on a daily basis (e.g., Instagram, Whatsapp, etc.) and how this relates to emoji selection in the G-Moji app. The continuous use of emojis throughout the day on platforms youth are already familiar with might reflect youths’ range of experienced emotions (e.g., moment by moment’), whereas the once a day selection of an emoji within G-Moji might rather capture youths’ reflective emotions (e.g., overall feeling).

In line with recent development in the field and to get a more accurate view on youth’s emotional state, future studies should, besides ecological momentary assessment (EMA), include digital phenotyping from mobile phone data collection, which shows a representation of a person’s digital patterns, that can help understand their mental health problems (29, 30). Passive data collection from personal digital devices, such as the smartphone, combined with daily measurement with emojis, may shift the focus from treatment to real-time prevention of (recurring) mental health problems.

## Ethics Statement

This study was approved by the Ethics Review Board of the University of Amsterdam, 2018-CDE-8836, ID 8836.

## Author Contributions

LV was the leading author. He designed the study, conducted the acquisition, and did the interpretation of the data. SR and EV supported with data collection and interpretation of the data. GS, MM, RV and conducted the analysis, whereby GS also helped with the interpretation and conceptualization of the total study. APo helped with the design. ES helped with the data-storage and data output. APe helped with the design of the study and the analyses. TR supported LV with the supervision of the study.

## Conflict of Interest Statement

The authors declare that the research was conducted in the absence of any commercial or financial relationships that could be construed as a potential conflict of interest.

## Appendix 1: Emojis

**Table:**

	Description	Emojis
*Positive emojis*		
1.	Funny, e.g. feeling yourself a little playful, childish in a positive way	
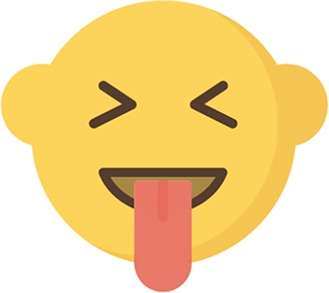
2.	Ecstatic, “super happy”	
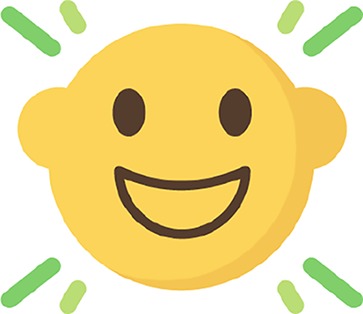
3.	Happy	
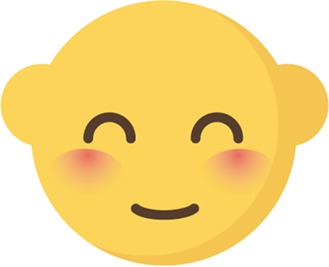
4.	Peaceful, relaxed,	
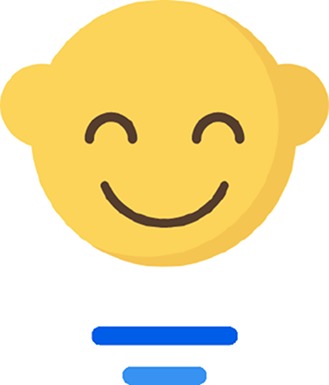
5.	Confident,	
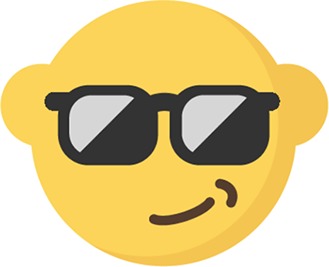
6.	In love, as in “in love with these shoes/person, etc”	
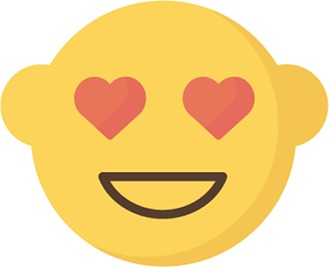
*Negative*		
7.	Confused	
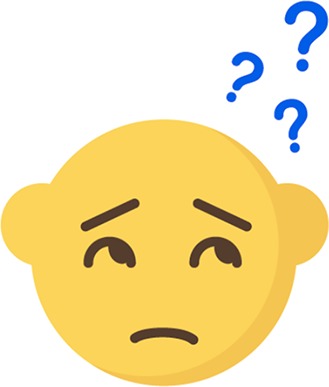
8.	Sad	
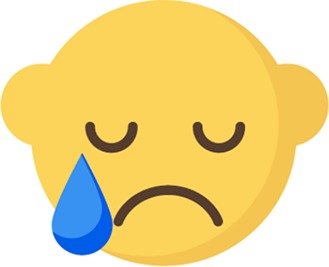
9.	Depressed	
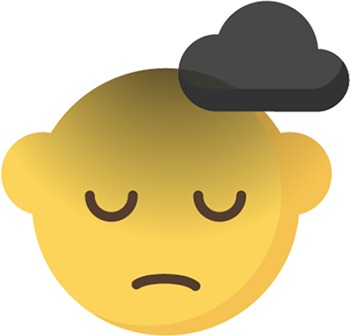
10.	Tired, exhausted	
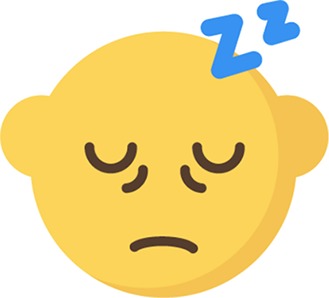
11.	Anxiety, scared	
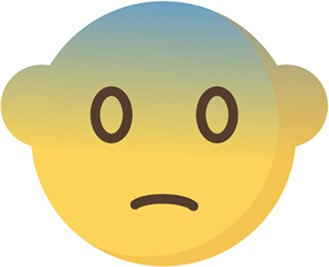
12.	Mad, angry	
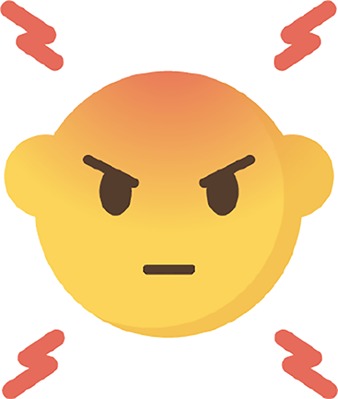
*Excluded*		
13.	Hopeless	
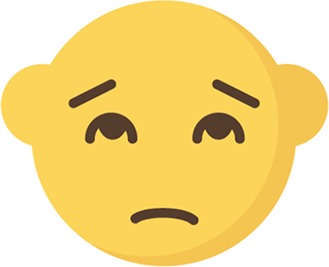
14.	Sick	
